# Supplementary material for: Structural Insight into a Yeast Maltase—The BaAG2 from Blastobotrys adeninivorans with Transglycosylating Activity
Source: J Fungi (Basel). 2021 Sep 29;7(10):816. doi: 10.3390/jof7100816 (PMC8539097; doi:10.3390/jof7100816)
Supplement: Supplementary file 1 [file jof-07-00816-s001.zip › jof-1384813-SI.pdf]

Article

Structural Insight into a Yeast Maltase—The *Ba*AG2 from *Blastobotrys adeninivorans* with Transglycosylating Activity

Karin Ernits [1], Christian Kjeldsen [2], Karina Persson [1], Eliis Grigor [3], Tiina Alamäe [3] and Triinu Visnapuu [3,*]

[1] Department of Chemistry, Umeå University, 90187 Umeå, Sweden; karin.ernits@umu.se (K.E.); karina.persson@umu.se (K.P.)
[2] Department of Chemistry, Technical University of Denmark, DK-2800 Kgs. Lyngby, Denmark; chkje@kemi.dtu.dk
[3] Institute of Molecular and Cell Biology, University of Tartu, 51010 Tartu, Estonia; eliis.grigor25@gmail.com (E.G.); tiina@alamae.eu (T.A.)
* Correspondence: triinu.visnapuu@ut.ee; Tel.: +372-7375013

Abstract: An early-diverged yeast, *Blastobotrys* (*Arxula*) *adeninivorans* (*Ba*), has biotechnological potential due to nutritional versatility, temperature tolerance, and production of technologically applicable enzymes. We have biochemically characterized from the *Ba* type strain (CBS 8244) the GH13-family maltase *Ba*AG2 with efficient transglycosylation activity on maltose. In the current study, transglycosylation of sucrose was studied in detail. The chemical entities of sucrose-derived oligosaccharides were determined using nuclear magnetic resonance. Several potentially prebiotic oligosaccharides with α-1,1, α-1,3, α-1,4, and α-1,6 linkages were disclosed among the products. Trisaccharides isomelezitose, erlose, and theanderose, and disaccharides maltulose and trehalulose were dominant transglycosylation products. To date no structure for yeast maltase has been determined. Structures of the *Ba*AG2 with acarbose and glucose in the active center were solved at 2.12 and 2.13 Å resolution, respectively. *Ba*AG2 exhibited a catalytic domain with a $(\beta/\alpha)_8$-barrel fold and Asp216, Glu274, and Asp348 as the catalytic triad. The fairly wide active site cleft contained water channels mediating substrate hydrolysis. Next to the substrate-binding pocket an enlarged space for potential binding of transglycosylation acceptors was identified. The involvement of a Glu (Glu309) at subsite +2 and an Arg (Arg233) at subsite +3 in substrate binding was shown for the first time for α-glucosidases.

Keywords: α-glucosidase; glycoside hydrolase; isomalto-oligosaccharides; acarbose; crystal structure; molecular replacement; nuclear magnetic resonance; erlose; isomelezitose; trehalulose

Citation: Ernits, K.; Kjeldsen, C.; Persson, K.; Grigor, E.; Alamäe, T.; Visnapuu, T. Structural Insight into a Yeast Maltase—The *Ba*AG2 from *Blastobotrys adeninivorans* with Transglycosylating Activity. *J. Fungi* **2021**, *7*, 816. https://doi.org/10.3390/jof7100816

Academic Editors: Paul Olusegun Bankole and Dietmar Schlosser

Received: 1 September 2021
Accepted: 24 September 2021
Published: 29 September 2021

Publisher's Note: MDPI stays neutral with regard to jurisdictional claims in published maps and institutional affiliations.

Copyright: © 2021 by the authors. Licensee MDPI, Basel, Switzerland. This article is an open access article distributed under the terms and conditions of the Creative Commons Attribution (CC BY) license (http://creativecommons.org/licenses/by/4.0/).

Supplementary Materials

Tables S1, S2, S3

Figures S1, S2, S3, S4, S5, S6, S7

Table S1. Substrates of α-glucosidases and their potential transglycosylation products. Oligosac- charides containing glucose and fructose residues, their composition and linkage types are shown. The substrates for *Ba*AG2 were determined in [1] and in this study. Sucrose isomers are shown in bold and sucrose moiety in trisaccharides is underlined.

Saccharide	Composition and linkage type	Description	Substrate for *Ba*AG2
Nigerose*	Glc-α1,3-Glc	Maltose-type disaccharide	ND
Maltose*	Glc-α1,4-Glc	Maltose-type disaccharide	+
Isomaltose*	Glc-α1,6-Glc	Isomaltose-type disaccharide	−
Trehalulose*	**Glc-α1,1-Fru**	Substrate; disaccharide	+
Sucrose	Glc-α1,2-Fru	Substrate; disaccharide	+
Turanose*	**Glc-α1,3-Fru**	Maltose-type disaccharide	+
Maltulose*	**Glc-α1,4-Fru**	Maltose-type disaccharide	+
Isomaltulose (palatinose)*	**Glc-α1,6-Fru**	Isomaltose-type disaccharide	−
Maltotriose	Glc-α1,4-Glc-α1,4-Glc	Maltose-type trisaccharide	+
Panose	Glc-α1,6-Glc-α1,4-Glc	Mixed-type trisaccharide	−
Esculose*	Glc-α1,3-<u>Glc-α1,2-Fru</u>	Maltose-type trisaccharide	ND
Erlose*	Glc-α1,4-<u>Glc-α1,2-Fru</u>	Maltose-type trisaccharide	+
Theanderose*	Glc-α1,6-<u>Glc-α1,2-Fru</u>	Isomaltose-type trisaccharide	ND
Melezitose*	Glc-α1,3-<u>Fru-β2,1-Glc</u>	Maltose-type trisaccharide	+
Isomelezitose*	Glc-α1,6-<u>Fru-β2,1-Glc</u>	Isomaltose-type trisaccharide	+

Glc — glucose; Fru — fructose; + hydrolysed by *Ba*AG2; − not a substrate for *Ba*AG2; ND — not determined.

* Products of *Ba*AG2 detected in this study by NMR from transglycosylation reaction on sucrose

Table S2. Data collection and refinement statistics of *Ba*AG2 structure determination.

	***Ba*AG2 + acarbose**	***Ba*AG2 + glucose**
**PDB ID**	**7P01**	**7P07**
**Data collection**		
Space group	P 1 21 1	P 1 21 1
Cell dimensions		
a, b, c (Å)	68.03 78.08 121.93	66.88 77.73 121.11
α, β, γ (°)	90 94.1 90	90 92.9 90
Resolution (Å)	48.14-2.12 (2.18-2.12)*	47.73-2.13 (2.19-2.13)*
*R*merge	0.023 (1.152)	0.097 (1.204)
I/σ*I*	7.68 (1.54)	8.61 (1.19)
Completeness (%)	98.9 (100.0)	98.9 (99.8)
Redundancy	3.55 (3.55)	3.55 (3.58)
**Refinement**		
Resolution (Å)	48.14-2.12	47.73-2.13
No. reflections	72345	69408
*R*work/*R*free	0.1842 /0.1952	0.2150 / 0.2318
No. non-hydrogen atoms	10177	9770
Protein	9345	9355
Ligand/ion	155	40
Water	677	375
B-factors	44.6	53.6
Protein	44.9	52.5
Ligand/ion	46.1	53.0
Water	40.1	80.2
R.m.s. deviations		
Bond lengths (Å)	0.019	0.017
Bond angles (°)	1.89	1.89

* Statistics for the highest-resolution shell is shown inparentheses.

Table S3. NMR assignment of Glc-α1,1-Fru-β2,1-Glc.

**Signal/Position**	**1**	**2**	**3**	**4**	**5**	**6**
α-Glc*p*-1,1-						
1H	4.953	3.488	3.683	3.371	3.596	3.709/3.732
13C	98.49	71.29	72.85	69.42	72.24	60.11
α-Glc*p*-1,2-						
1H	5.371	3.479	3.670	3.379	3.772	3.713/3.778
13C	92.54	71.024	72.548	69.23	72.31	60.48
β-Fru*f*-2,1-						
1H	3.607/3.816	-	4.210	3.989	3.861	3.747
13C	65.99	103.58	76.46	73.68	81.24	62.16

```
BaAG2_CBS 8244    MVLGFFKKEQERNTAWWREGTVYQVYPSSFKDSNGDGIGDIPGIISKLDYIKSVGTDIVW    60
BaAG2_TMCC 70007  MVLGFFKKEQERNTAWWREGTVYQVYPSSFKDSNGDGIGDIPGIISKLDYIKSVGTDIVW    60
BaAG2_LS3         MVLGFFKKEQERNTAWWREGTVYQVYPASFKDSNGDGIGDIPGIISKLDYIKGVGADIVW    60
                  ***************************:************************.**:****

BaAG2_CBS 8444    LSPHYKSPQVDMGYDISDYKAIHEPYGTFDDCIKLIQECHDRGLKIIFDLVVNHTSDQHE    120
BaAG2_TMCC 70007  LSPHYKSPQVDMGYDISDYKAIHEPYGTFDDCIKLIQECHNRGLKIIFDLVVNHTSDQHE    120
BaAG2_LS3         LSPHYKSPQIDMGYDISDYKAIHEPYGTFDDCIKLIQECHDRGLKIIFDLVVNHTSDQHE    120
                  *********:******************************:*******************

BaAG2_CBS 8244    WFKQSRSSKSNAKRDWYIWKPAKYDEQGNRIPPNNWESYFGGSAWEWDEETQEYYLHLFA    180
BaAG2_TMCC 70007  WFKQSRSSKSNPKRDWYIWKPAKYDEQGNRIPPNNWESYFGGSAWEWDEETQEYYLHLFA    180
BaAG2_LS3         WFKQSRSSKSNPKRDWYIWKPAKYDEQGNRIPPNNWESYFGGSAWEWDEQTQEYYLHLFA    180
                  *********** *************************************:**********

BaAG2_CBS 8244    KEQPDINWRNLQAREAIYKDAILFWLDRGIDGFRIDTVQIYSKPEDFPDAPERVPGQKLQ    240
BaAG2_TMCC007     KEQPDINWRNLQAREAIYKDAILFWLDRGIDGFRIDTVQIYSKPEDFPDAPERVPGQKLQ    240
BaAG2_LS3         KEQPDINWRNLQAREAIYKDAILFWLERGIDGFRIDTVQIYSKPEDFPDAPERVPGQKLQ    240
                  **************************:*********************************

BaAG2_CBS 8244    NPSIIVETGPQLHEILQEMNRKAFSKYDIMTVGEGSPPSLEKTLEYVSSSRHEIDMLFSF    300
BaAG2_TMCC 70007  NPSIIVETGPQLHEILQEMNRKAFSKYDIMTVGEGSPPSLEKTLEYVSSSRHEIDMLFSF    300
BaAG2_LS3         NPSIIVETGPQLHEILQEMNRKAFSKYDIMTVGEGSPPSLEKTLEYVGSSRHEIDMLFSF    300
                  ***********************************************.************

BaAG2_CBS 8244    DFGRLDHREIAHTLNDIDMVEMKETVYRWQRLVGKTDGWTTFFLENHDSGRSISRFASDA    360
BaAG2_TMCC 70007  DFGALDHREIAHTLNDIDMVEMKETVYRWQRLVGKTDGWTTFFLENHDSGRSISRFASDA    360
BaAG2_LS3         DFGRLDHSEIAHTLNDIDMVEMKETVYRWQRLVGKTDGWTTFFLENHDSGRSISRFASDA    360
                  *** *** ****************************************************

BaAG2_CBS 8244    TPEARNRSTKFLAVLQATMSGTLYLYQGQEIGIPNLPDEVPIEEYKDVNSINYYNAVKED    420
BaAG2_TMCC 70007  TPEARTRSTKFLAVLQATMSGTLYLYQGQEIGIPNLPDEVPIDEYKDVNSINYYNAVKED    420
BaAG2_LS3         TPEARTRSTKFLAVLQATMSGTLYLYQGQEIGIPNLPDEVPIDEYKDVNSINYYNAVKED    420
                  *****.************************************:*****************

BaAG2_CBS 8244    TKNDPDALKKAKKYLQKVARDHARSPMQWDASKHSGFTDGEPWMIVNPIYPEVNVAAQEW    480
BaAG2_TMCC 70007  TNNDPDALKKAKKYLQKVARDHARSPMQWDASKHSGFTEGEPWMIVNPIYPEVNVAAQEW    480
BaAG2_LS3         TKHDPEALKQAKKYLQKVARDHARSPMHWDASKHSGFTDGEPWMIVNPIYPEVNVAAQEW    480
                  *::**:***:*****************:**********:*********************

BaAG2_CBS 8244    DADSGLNFWRHILRFRRQHKDVMVYGTLEVLDKENTKVFTFTKASESGRTFLVALNFTDK    540
BaAG2_TMCC 70007  DADSGLNFWRHILRFRREHKDVMVYGTLEILDKENTKVFTYTKASESGRTFLVALNFTDK    540
BaAG2_LS3         DADSGLNFWRHILKFRRQHKDTMVYGKLEVLDKENTKVFTFTKTSESGRTILVALNFTDK    540
                  *************:***:***.****.**:**********:**:******:*********

BaAG2_CBS 8244    AVDYDVDQFASKSTLLLSSIGNVHHKSGSLQPLEAVIFEI 580
BaAG2_TMCC 70007  AVDYNVNQFASKSTLLLSSIGNVHHQSGSLQPLEAVIYEI 580
BaAG2_LS3         AVDYNVNQFASKSTLLLSSIGNVHHQSGSLQPLEAVIYEI 580
                  ****:*:******************:***********:**
```

Figure S1. Clustal Omega alignment of *Ba*AG2 homologues from strains of three *Blastobotrys* (*Arx- ula*) sp. BaAG2_CBS 8244, a maltase from the *Ba* type strain which is a source of the *Ba*AG2 in the current work and our earlier studies [1,2]. BaAG2_TMCC 7007, a putative maltase of a *Ba* strain isolated from fermented Pu-erh tea [3]; BaAG2_LS3, a putative maltase of the first strain of *Blastobotrys* sp. with sequenced genome [4] that is currently allocated to *Blastobotrys raffinosifer- mentans* [5]. Amino acids constituting the catalytic triad are shown by cyan background and those interacting with substrate at −1, +1, +2 and +3 subsites are marked using grey background. Clustal consensus is marked below the sequence indicating conservation — * positions with fully con- served residue; : positions with residues of strongly similar properties; . positions with residues of weakly similar properties.

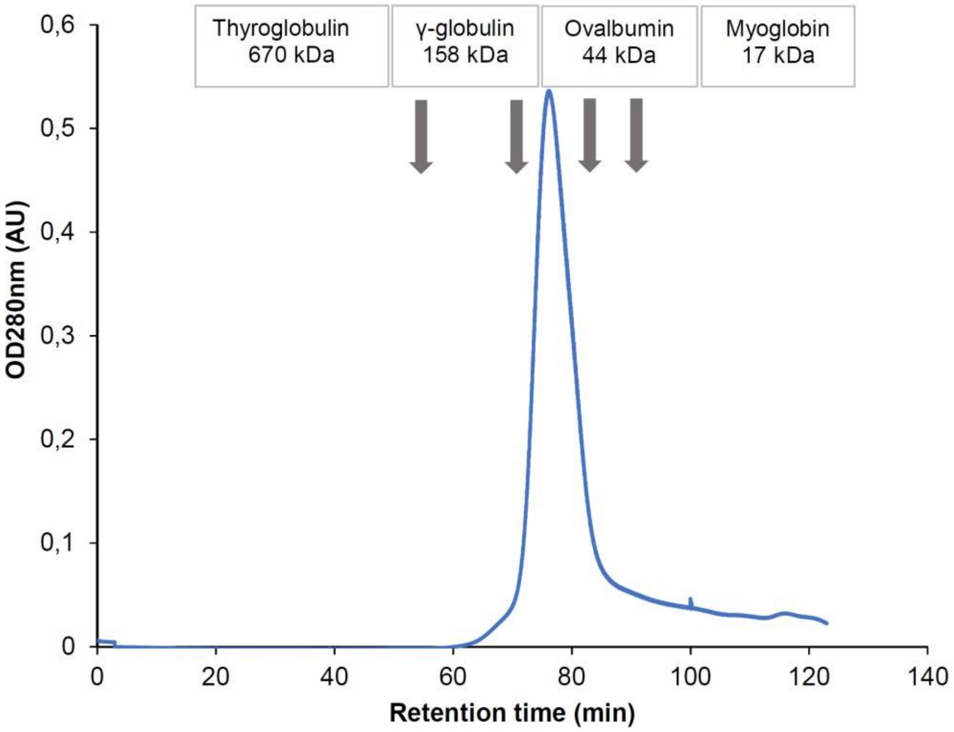

Figure S2. The chromatogram of size-exclusion chromatography of *Ba*AG2. The chromatography was performed on Hiload 16/60 Superdex 200 pg column (GE Healthcare, Uppsala, Sweden) as shown in chapter 2.1. in the main text. Gel filtration standard from Bio-Rad (Hercules, CA, USA) was used for calibration. The reference proteins with their molecular weights are shown and re- spective retention times are indicated by arrows. According to the calibration curve, the peak max- imum of *Ba*AG2 corresponds to 75.7 kDa.

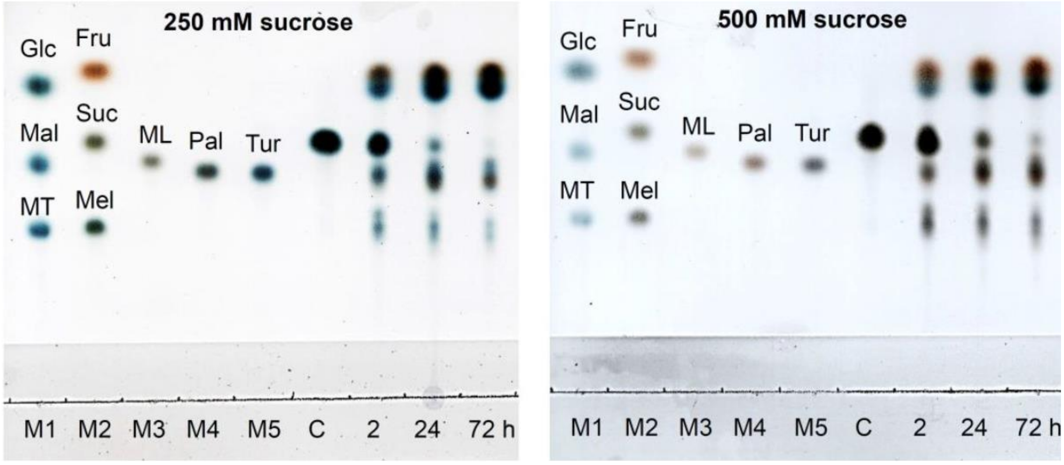

Figure S3. TLC analysis of transglycosylation on 250 mM or 500 mM sucrose by *Ba*AG2. The re- sults on quantification of saccharides by HPLC is shown on Figure 2 in the main text. The final concentration of the enzyme in the reaction mixture was 20 µg/mL. The reactions were carried out at 30 °C up to 72 h as shown in chapter 2.4. in the main text. Samples were 5 times diluted in mQ water before applying to TLC plates in 0.5 µL spots. The sugars were separated by TLC and visu- alized as shown in chapter 2.6. in the main text. Marker sugars were analysed as references. M1: Glc – glucose (30 mM), Mal – maltose (10 mM), MT – maltotriose (10 mM); M2: Fru – fructose (30 mM), Suc – sucrose (10 mM), Mel – melezitose (10 mM); M3: ML – maltulose (10 mM); M4: Pal – palatinose (isomaltulose, 10 mM); M5: Tur – turanose (10 mM). C – Control without enzyme but with 0.5% BSA and substrate (sucrose) incubated 72 h alongside to the samples. The compositions of reference sugars are shown in Table S1.

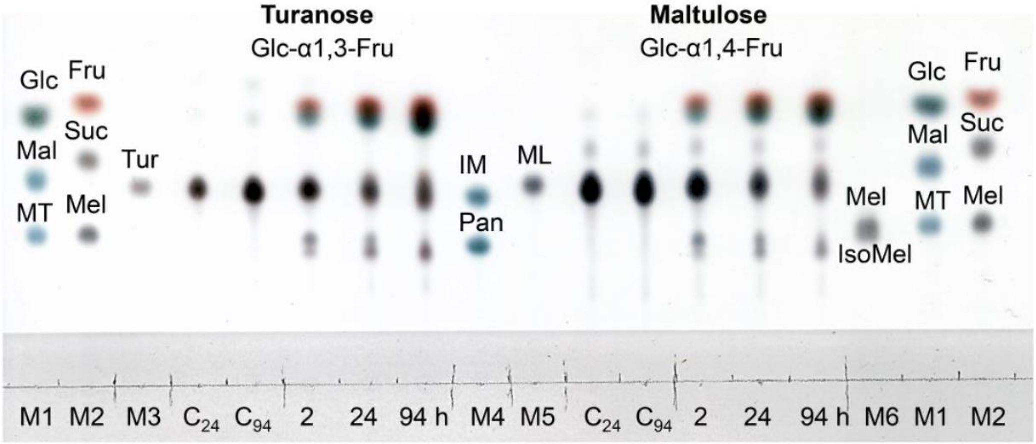

Figure S4. Transglycosylation assay of 500 mM turanose and maltulose by *Ba*AG2. The final con- centration of the enzyme was 20 μg/mL. The reactions were carried out at 30 °C up to 94 h simi- larly as shown in chapter 2.4. in the main text. Samples were 5 times diluted in mQ water before applying to TLC plates in 0.5 μL spots. The sugars were separated by TLC and visualized as shown in chapter 2.6. in the main text. M1: Glc – glucose (30 mM), Mal – maltose (10 mM), MT – maltotriose (10 mM); M2: Fru – fructose (30 mM), Suc – sucrose (10 mM), Mel – melezitose (10 mM); M3: Tur – turanose (10 mM); M4: IM – isomaltose (10 mM), Pan – panose (10 mM); M5: ML – maltulose (10 mM); M6: Mel – melezitose (10 mM), IsoMel – isomelezitose (*Op*MAL1 DP 3 prod- uct); C_{24} and C_{94} – Control without enzyme but with 0.5% BSA and substrate (turanose or maltu- lose) incubated alongside to the transglycosylation samples up to 24 h and 94 h, respectively. The compositions of reference sugars are shown in Table S1

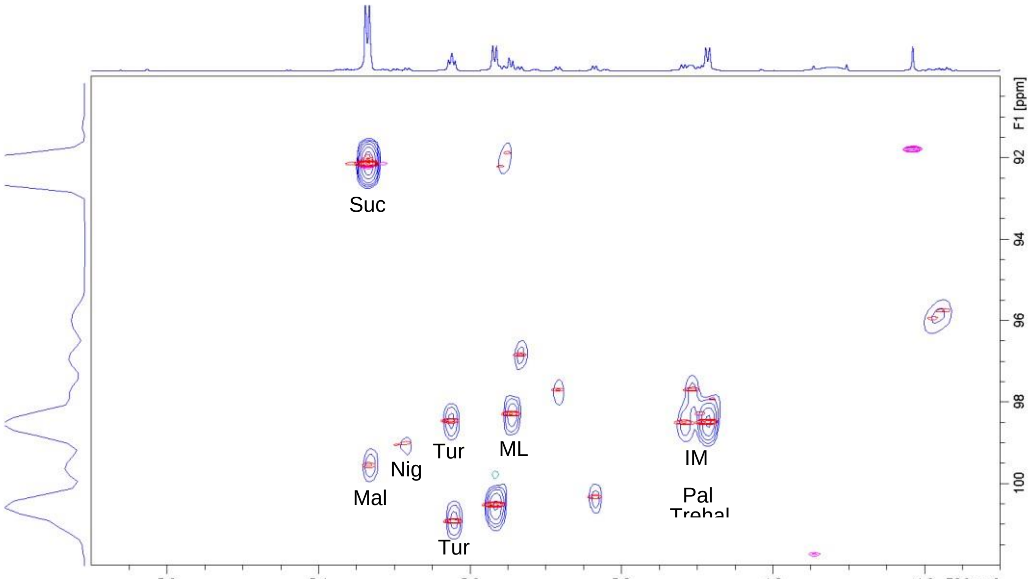

Figure S5. HSQC spectrum of the anomeric region (blue) overlaid with narrow HSQC (red) of the DP 2 sample prepared using 500 mM sucrose as substrate. The NMR spectra were recorded as shown in chapter 2.5. and analysed as described in chapter 3.4. in the main text. The ^{1}H projection is from a separate experiment. The labels of non-reducing anomeric signals are as follows: Suc – sucrose, Mal – maltose, Nig – nigerose; Tur – turanose, ML – maltulose, IM – isomaltose, Pal – palatinose (isomaltulose), Trehal – trehalulose. The compositions of sugars are shown in Table S1.

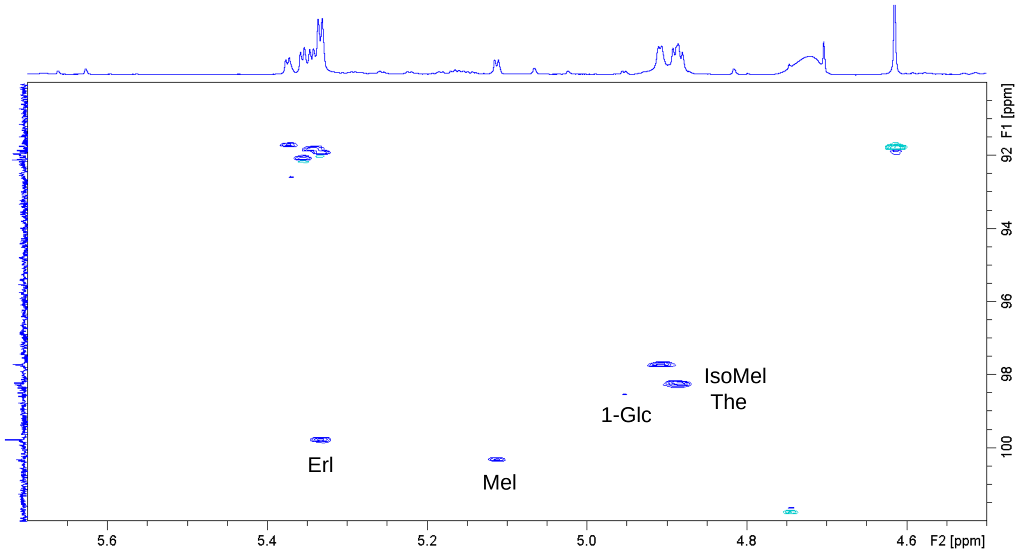

Figure S6. Narrow HSQC spectrum of the DP 3 sample prepared using 500 mM sucrose as sub- strate. The NMR spectra were recorded as shown in chapter 2.5. and analysed as described in chapter 3.4. in the main text. The ^{1}H and ^{13}C projections are from separate experiments. The labels are of non-reducing anomeric signals not arising from sucrose (Glc-α1,2β-Fru), and are ordered by descending ^{1}H chemical shift as follows: Erl – erlose, Mel – melezitose, 1-Glc – 1-O^{F}-glycosyl-su- crose, IsoMel – isomelezitose, The – theanderose.

Journal of *Fungi*

MDPI

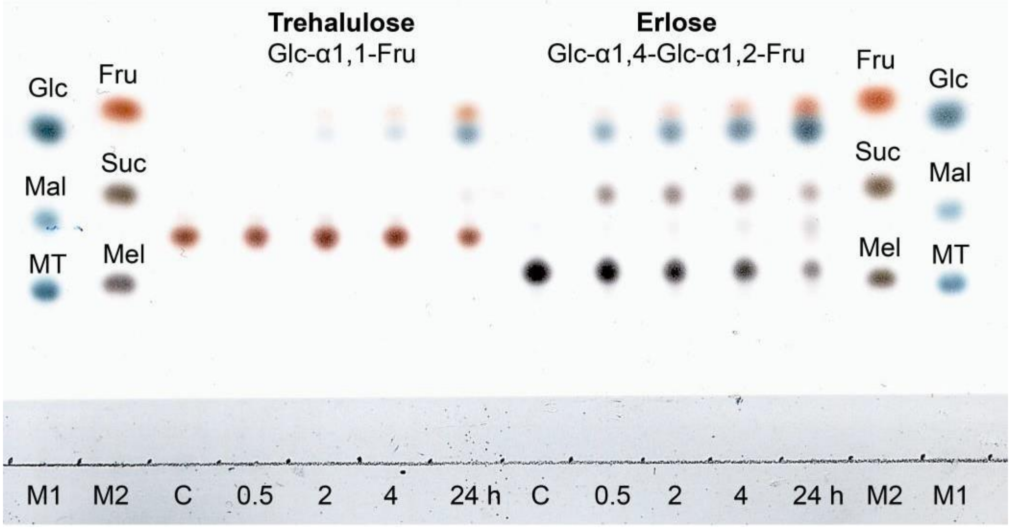

Figure S7. TLC analysis of *Ba*AG2 reaction products from 50 mM trehalulose and erlose. The final concentration of the enzyme was 3 µg/mL in the reaction mixture. The reactions were carried out at 30 °C up to 24 h similarly as in chapter 2.4. in the main text. Samples were 2 times diluted in mQ water before applying to TLC plates in 0.5 µL spots. The sugars were separated and visualized as shown in chapter 2.6. in the main text. M1: Glc – glucose (30 mM), Mal – maltose (10 mM), MT – maltotriose (10 mM); M2: Fru – fructose (30 mM), Suc – sucrose (10 mM), Mel – melezitose (10 mM); C – Control without enzyme but with 0.5% BSA and substrate (trehalulose or erlose, respec- tively) incubated 24 h alongside to the samples.

References

1. Visnapuu, T.; Meldre, A.; Põšnograjeva, K.; Viigand, K.; Ernits, K.; Alamäe, T. Characterization of a Maltase from an Early-Diverged Non-Conventional Yeast *Blastobotrys adeninivorans*. *Int. J. Mol. Sci.* **2020**, *21*, 297, doi:10.3390/ijms21010297.
2. Viigand, K.; Põšnograjeva, K.; Visnapuu, T.; Alamäe, T. Genome Mining of Non-Conventional Yeasts: Search and Analysis of MAL Clusters and Proteins. *Genes* **2018**, *9*, 354, https://doi.org/10.3390/genes9070354.
3. Tian, F.; Shi, J.; Li, Y.; Gao, H.; Chang, L.; Zhang, Y.; Gao, L.; Xu, P.; Tang, S. Proteogenomics Study of *Blastobotrys adeninivorans* TMCC 70007-A Dominant Yeast in the Fermentation Process of Pu-Erh Tea. *J. Proteome Res.* **2021**, *20*, 3290–3304, doi:10.1021/acs.jproteome.1c00205.
4. Kunze, G.; Gaillardin, C.; Czernicka, M.; Durrens, P.; Martin, T.; Böer, E.; Gabaldón, T.; Cruz, J.A.; Talla, E.; Marck, C.; *et al.* The Complete Genome of *Blastobotrys (Arxula) adeninivorans* LS3 - a Yeast of Biotechnological Interest. *Biotechnol. Biofuels* **2014**, 7, 66, doi:10.1186/1754-6834-7-66.
5. Thomas, S.; Sanya, D.R.A.; Fouchard, F.; Nguyen, H.-V.; Kunze, G.; Neuvéglise, C.; Crutz-Le Coq, A.-M. *Blastobotrys adeninivo- rans* and *B. raffinosifermentans*, Two Sibling Yeast Species Which Accumulate Lipids at Elevated Temperatures and from Diverse Sugars. *Biotechnol. Biofuels* **2019**, *12*, 154, doi:10.1186/s13068-019-1492-x.
